# Parental Perception of childhood obesity: Alechia Van Wyk

**DOI:** 10.1093/eurpub/ckac131.432

**Published:** 2022-10-25

**Authors:** A Van Wyk

**Affiliations:** Middlesex University, Hendon, UK

## Abstract

**Background and aims:**

Childhood obesity rate continues to grow in the UK with children from deprived backgrounds being at greater risk. While a large part of the literature focuses on the determinants of child obesity less is known about the factors that can lead a parent to misclassify their child’s weight status. The aim of this study was to investigate parental perception of their child’s weight status, compared to their measured weight.

**Methods:**

Height, weight, and waist circumference were measured for 232 school children enrolled in Year 2 and Year 5 during the academic year 2019-2020 from ten schools in Barnet Council, London, UK. In addition, data were collected from parents on their child’s weight status and perception, health beliefs, eating habits, exercise habits, family anthropometric and health information. Information from 110 mothers and 17 fathers was retrieved. The WHO growth charts were used to classify the children’s BAZ scores.

**Results:**

Results show that 46% of the parents classified their child’s weight status incorrectly, they either overestimated or underestimated their child’s body weight and/or shape. Specifically, 52% of parents of overweight/obese children reported their children as normal weight. Factors associated with parents’ child weight status misclassification child’s age (p = .771), ethnicity (p = .445), parents’ education (p = .227), and marital status (p = 0.07).

**Conclusions:**

Parental perception of child’s weight status has important implications in terms of changes in household eating behaviours and attitudes. Understanding the key factors affecting parental perception is of paramount importance when developing interventions and policies aimed at tackling child obesity.

**Key messages:**

• Parents classify their children’s weight status incorrectly, making it hard for policy change to be effective.

• Policy change can only happen if parents play an active role in understanding and perceiving if their child is overweight or obese.

